# Ready to Change: Attitudes of an Elderly CKD Stage 3–5 Population towards Testing Protein-Free Food

**DOI:** 10.3390/nu12113519

**Published:** 2020-11-16

**Authors:** Elisa Longhitano, Tiziana Trabace, Antioco Fois, Antoine Chatrenet, Maria Rita Moio, Francoise Lippi, Jerome Vigreux, Coralie Beaumont, Domenico Santoro, Massimo Torreggiani, Giorgina Barbara Piccoli

**Affiliations:** 1 Renal Unit, Department of Clinical and Experimental Medicine, University of Messina, 98100 Messina, Italy; elisa.longhitano@libero.it (E.L.); dsantoro@unime.it (D.S.); 2 Nephrology and Dialysis, Centre Hospitalier Le Mans, Avenue Roubillard 194, 72000 Le Mans, France; tizi.trb87@gmail.com (T.T.); antiocofois@gmail.com (A.F.); antoine.chatrenet@gmail.com (A.C.); mariaritamoio@gmail.com (M.R.M.); maxtorreggiani@hotmail.com (M.T.); 3 Service of Dietetics, Centre Hospitalier Le Mans, Avenue Roubillard 194, 72000 Le Mans, France; flippi@ch-lemans.fr (F.L.); jvigreux@ch-lemans.fr (J.V.); cbeaumont@ch-lemans.fr (C.B.); 4 Dipartimento di Scienze Cliniche e Biologiche, University of Torino, 10100 Torino, Italy

**Keywords:** chronic kidney disease, protein restriction, protein-free food, nutrition in CKD, low-protein diet

## Abstract

The recent Kidney Disease Outcomes Quality Initiative (K-DOQI) guidelines suggest an early start of protein restriction, raising issues on willingness to change dietary habits. The aim of this exploratory real-life study was to report on a test of dietary products (protein-free, not previously available in France) in a large, mainly elderly, chronic kidney disease (CKD) population (220 patients, median age: 77.5 years, Charlson comorbidity index (CCI): seven, malnutrition inflammation score (MIS): five, estimated glomerular filtration rate (eGFR): 26 mL/min), also as a means to tailor further implementation strategies. Forty-nine patients (22.28%) were considered to be poor candidates for the trial (metabolically unstable or with psychological, psychiatric or logistic barriers); of the remaining 171, 80.70% agreed to participate. Patients to whom the diet was not proposed had lower eGFR and higher comorbidity (eGFR 21 vs. 27 *p* = 0.021; MIS six vs. four *p*: <0.001). Patients who refused were 10 years older than those who accepted (83 vs. 73 years *p* < 0.001), with a higher CCI (eight vs. seven *p* = 0.008) and MIS (five vs. four *p* = 0.01). In the logistic regression, only age was significantly associated with refusal to participate (Odds ratio (OR): 5.408; 95% CI: 1.894 to 15.447). No difference was found according to low/intermediate/high frequency of weekly use of protein-free food. Our study suggests that most of the patients are ready to test new diet approaches. Only old age correlated with refusal, but frequency of implementation depended on individual preferences, underlying the importance of tailored approaches to improve adherence.

## 1. Introduction

Chronic kidney disease (CKD) is a complex heterogeneous disease in terms of pathogenesis, clinical manifestations, speed of progression, and development of complications. However, despite its heterogeneity, when over 50–70% of the renal parenchyma is damaged, a progressive loss of kidney function is usually observed regardless of the initial cause. Slowing the progression of renal damage becomes crucial in earlier phases, while metabolic stabilization in late CKD phases sometimes makes it possible to postpone dialysis. For this purpose, nutritional management, in particular protein restriction, plays a role in protecting kidney function [1,2,3,4,5,6,7,8,9,10,11,12,13,14,15,16]. In fact, the new guidelines of the Kidney Disease Outcomes Quality Initiative (K-DOQI) suggest protein restriction should be introduced early, when possible in stage three, with a profile adapted to each patient, provided that the patients are metabolically stable and free from acute diseases—especially if these diseases potentially induce wasting [17].

The understanding of the link between uremic syndrome and exogenous protein catabolism can be traced back to the nineteenth century; however, only in the Sixties, mainly thanks to Giordano, Giovannetti and Maggiore, low-protein diets were systematically prescribed for patients with advanced renal insufficiency [18,19,20]. While several dietary approaches were and are still available, Maggiore is mainly behind the definition of low-protein diets based upon the substitution of usual bread and pasta with protein-free equivalents. These products are commonly used in Italy where they are fully reimbursed and are a cornerstone of dietary CKD management [18,21,22,23].

The dietary management of CKD encountered different fortunes over the subsequent decades but it is now increasingly recognized as fundamental for delaying the need for renal replacement therapy [7,10,11,12,24,25,26]. The latest K-DOQI guidelines on nutrition in patients with CKD strongly recommend (evidence level: 1A) protein restriction not only to reduce the risk of end-stage renal disease but also death [17]. When patients following such diets are carefully assessed, the risk of malnutrition is now far lower than in the past [2,24,27,28,29,30].

However, following a low-protein diet is difficult and elderly patients, who represent the vast majority of our population, are often considered to be “resistant” to change [21].

In this context, we decided to carry out this study to explore patients’ willingness to change their diet when offered new options. Enrolling patients in a test of protein-free food that had not previously been available in France allowed us to evaluate attitudes towards dietary changes in a large cohort of high-comorbidity elderly patients with advanced CKD. 

## 2. Materials and Methods

### 2.1. Study Setting

The study was conducted in the advanced chronic kidney failure unit (UIRAV Unite pour l’Insuffisance Rénale AVancée) that opened in November 2017 in the Centre Hospitalier Le Mans (CHM) in France. CHM is one of the largest non-university hospitals in France with a referral area of about 500,000 inhabitants. UIRAV provides care for outpatients with advanced kidney disease or with specific clinical needs (including pregnancy). Three nutritionists and two nephrologists work in the unit, providing integrated follow-up including dietary advice and nutritional evaluation [31]. 

### 2.2. Study Population

The study population encompassed all the 220 stage 3 to stage 5 patients seen by UIRAV between 24 March 2019 and 24 June 2020. In keeping with the KDOQI guidelines, a tailored protein reduction policy was recommended for patients in stages 3–5 without contraindications (including malnutrition, or being metabolically unstable, the latter encompassing the main conditions associated with short life expectancy) [17]. 

Malnutrition was clinically defined, corresponding overall to subjective global assessment (SGA) score C or to switch between SGA scores A and B, while diets were prescribed in stable patients rated as SGA B. Short life expectancy was likewise considered empirically as a life expectancy of less than 3 months.

Patients were offered protein-free products in the context of the Pro-Re-Pro (Protein restriction for retarding CKD Progression) study, prospectively testing the feasibility, implementation, and results of a tailored approach to low-protein diets in CKD stages 3–5. No age limit was posed, but the unit gathers patients with advanced CKD, particularly if at high morbidity, notably cardiovascular. This explains why the median age is so high, in a setting where the median age at the start of dialysis is above 70 years. 

The reasons why some patients were not asked to participate in the test and patients’ reasons for deciding not to try the new products were recorded, while for patients who agreed to participate, compliance, barriers and satisfaction were recorded. 

All recruited patients had performed at least one previous nephrology visit in the unit, or in the other units ran by the same team, and 90% of them had visited more than once, in keeping with the policy of progressively prescribing dietary changes, according to a personalized approach. The median interval between the first enrollment in the UIRAV and the proposal of the test was 244 days.

The variables analyzed include gender, ethnicity, age, body mass index (BMI), dietary prescriptions at the time of the test, serum creatinine, estimated glomerular filtrate rate (calculated by means of the Chronic Kidney Disease Epidemiology Collaboration equation—CKD-EPI) [32], CKD stage, proteinuria, Charlson comorbidity index (CCI), subjective global assessment (SGA) index, malnutrition inflammation score (MIS), and, for patients who agreed to participate in the study, the Word Health Organisation Quality of Life Sort questionnaire (WHOQOL-BREF) was completed at baseline and a custom-made satisfaction questionnaire was completed after testing [33,34,35].

Chronic kidney disease was defined as is customary, according to the K-DOQI guidelines, based on the presence of either decreased kidney function (estimated glomerular filtration rate (eGFR) < 60 mL/min) or other morphological or urinary alterations lasting for at least 3 months. All the patients enrolled in the study had been classified as having CKD with at least one confirmatory eGFR.

### 2.3. Design of the Study and Choice of the Products

During their routine nephrology visit, either the caregiver nephrologist or a resident of the unit asked patients if they were interested in testing protein-free products, including at least one of four types of bread (sandwich bread, baguette, country loaf, crisp bread), two types of pasta (anellini and penne) and two types of cookies (fruit bars and wafers). The clinical team is small and consists of two nephrologists and one resident, working in close partnership, and thus ensuring homogeneous information and recruitment policy.

They were given portions of the bread, pasta, and cookies for 4 meals. One portion was calculated as 80 g of pasta and 50 g of bread, 2–4 biscuits and one snack, respectively. All products were given free of charge and no biochemical test was added to the usual routine; all biochemical tests are reimbursed for patients with advanced CKD in the French system. 

After testing the products, at their next visit or during a telephone interview (1–3 months after the test), patients were asked to complete the satisfaction questionnaire. 

### 2.4. Satisfaction Questionnaire

The questionnaire consisted of 3 parts:

- Assessment of satisfaction with the products tested, based on a 1–10 visual analogue scale;
- Identification of the frequency with which new products could replace products they normally ate;
- Semi-structured questions on possible barriers (price, variety of products, therapeutic efficacy, ease of supply, reimbursement, taste) to their use.

Readiness to change was scored as: “low” (max two portions/week), “moderate” (one portion/day), and “high” (at more than one portion/day). 

### 2.5. Statistical Analysis

Statistical analysis was performed with SPSS v.14 (IBM corp., Armonk, NY, USA) and JASP v.0.11.1 (JASP Team, Amsterdam, The Netherlands). Descriptive analysis was conducted as appropriate; the Shapiro–Wilk test was used to verify the normality distribution of the data, and the Levene’s test was used to verify homoscedasticity. According to the distribution of the data, the unpaired *t*-test was used to compare two groups (e.g., proposed vs. not proposed) or the ANOVA Test was used to compare n-groups (e.g., low, moderate and high willingness to change), otherwise the Mann–Whitney test was applied to compare two groups or the Kruskal–Wallis test was used for additional comparisons. Category results were presented as size with their percentage and compared with the chi-squared test or Fisher’s exact test for small subgroups. 

Statistical analysis was performed with SPSS v.14 (IBM corp., Armonk, NY, USA) and JASP v.0.11.1 (JASP Team, Amsterdam, The Netherlands). Descriptive analysis was conducted as appropriate; the Shapiro–Wilk test was used to verify the normality distribution of the data, and the Levene’s test was used to verify homoscedasticity. According to the distribution of the data, the unpaired *t*-test was used to compare two groups (e.g., proposed vs. not proposed) or the ANOVA Test was used to compare n-groups (e.g., low, moderate and high willingness to change), otherwise the Mann–Whitney test was applied to compare two groups or the Kruskal–Wallis test was used for additional comparisons. Category results were presented as size with their percentage and compared with the chi-squared test or Fisher’s exact test for small subgroups. 

Logistic regressions were performed to test the following two outcomes: refusal to try products and low willingness to change. The following explanatory variables were used: age (dichotomized ≥80 years), CCI (dichotomized ≥7), SGA score (B-C vs. A) and gender (females vs. males). The MIS score was not employed because of collinearity with Charlson and SGA scores. The variables were chosen either because they were clinically relevant (gender, SGA, Charlson) or because they were significant in the descriptive analysis (age).

A two-tailed alpha risk at 5% was considered statistically significant.

### 2.6. Ethical Issues

The study was conducted in accordance with the Declaration of Helsinki. 

The study was a part of the Pro-Re Re-Pro study. This is a prospective, monocentric, non-randomized study on the implementation of nutritional approaches to delay dialysis. The study started on 1 April 2019 and will end on 1 April 2023. The protocol of the Pro-Re Re-Pro study was approved by the ethics committee of the University Hospital de Toulouse (Comité de Protection des Personnes (CPP)) on 7 December 2018. Informed consent was obtained from each patient and there was anonymous management of clinical data.

## 3. Results

### 3.1. Baseline Data

**Figure 1:**
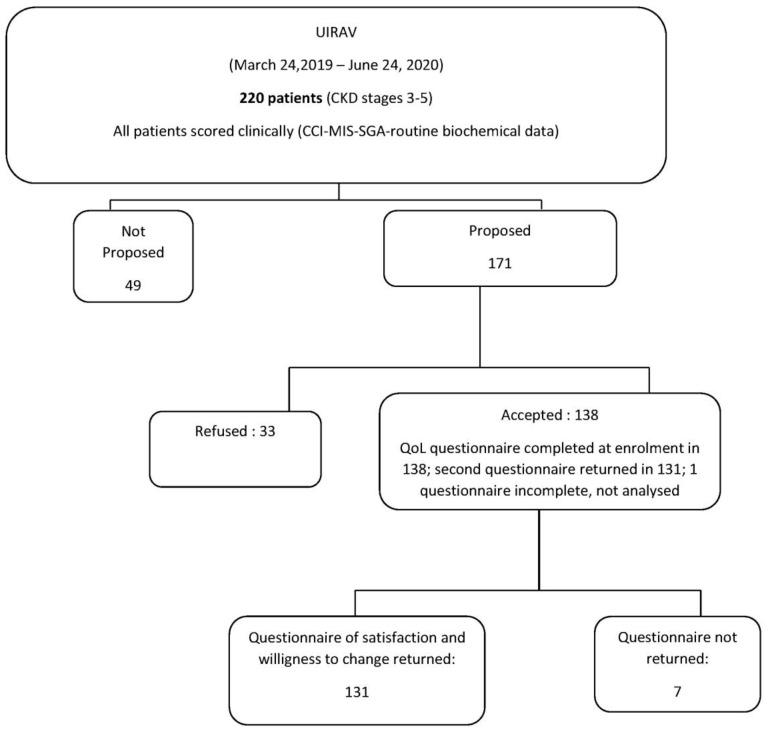
Study flow chart. CKD: chronic kidney disease; UIRAV: Unit for the follow-up of advanced CKD; CCI: Charlson Comorbidity Index; MIS: Malnutrition Inflammation Score; SGA: Subjective Global Assessment.

Figure 1 presents the study flow chart. Participation in the study was not proposed to 49 patients for reasons that included cognitive impairment (13/49), imminent dialysis (8/49) and short life expectancy—metabolic instability (10/49), other reasons (18/49) included follow-up based in another setting and language barriers. Participation in the study was proposed to 171 patients (77.73%). Of these, 33 (19.30%) refused and 138 (80.70%) agreed to participate. One hundred thirty-one (96.32%) satisfaction questionnaires were returned and only seven were not (two because the patients had died and five for various causes); one questionnaire returned was too incomplete to be analyzed. 

Table 1 reports the characteristics of the population divided as shown in the flow chart into four subsets (proposed vs. not proposed; agreed vs. refused). Kidney function improved in two patients who reached an eGFR of 61 and 68 mL/min, respectively, shifting from CKD stage 3a to stage two at recruitment, and thus explaining the eGFR range reported in Table 1.

Patients to whom the trial was not proposed had lower eGFR and higher comorbidity, in keeping with the shared indication not to consider metabolically unstable patients for protein restriction policies [17]; furthermore, about two-thirds of these patients were on an unrestricted diet (no restriction, or normalization of protein intake at 0.8 g/kg/day).

Conversely, patients who refused to try the new products were about 10 years older than those who accepted and, consequently, they displayed significantly higher CCI and MIS scores, and a lower prevalence of cases without signs of malnutrition.

### 3.2. Willingness to Change and Satisfaction with the Test

Table 2 reports the characteristics of the patients with respect to their willingness to change their diets, scored according to the frequency of integration of the suggested products in their weekly meal plans. As shown in Table 2, no significant difference was found in the three groups with the parameters analyzed. 

**Figure 2:**
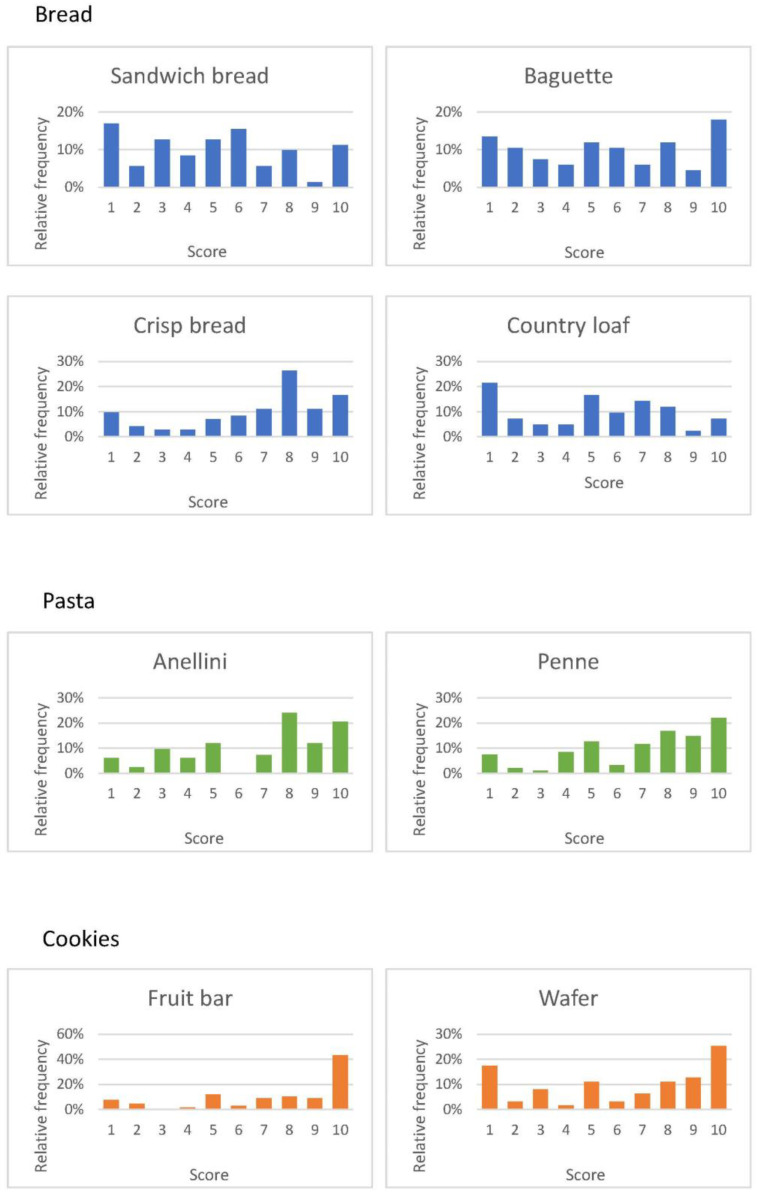
Satisfaction score from 1 (did not like) to 10 (liked very much) for the different food choice.

The product patients liked best were the fruit bars, with a median score of nine on a 1–10 visual analogue scale; pasta ranked second with a median of eight, while the bread products were not given high scores (Figure 2). 

Of the barriers analyzed (price, variety of products, therapeutic efficacy, ease of supply, reimbursement, taste), the most important was taste, followed by logistic barriers (price, reimbursement) and therapeutic efficacy. Ease of supply and variety were perceived as less relevant.

### 3.3. Logistic Regression Analysis: Refusal to Participate in the Test and Low Willingness to Change

Age was the only covariate associated with patients’ willingness to test new products. Patients aged ≥80 years had an odds-ratio of 5.891 (unadjusted) and 5.408 (adjusted) in refusing to test new food products compared to younger patients (Table 3). 

Conversely, none of the variables tested were associated with the frequency with which patients indicated they would be willing to integrate the new food into their diet (Table 4).

## 4. Discussion

The newly released guidelines on dietary management of CKD underline the importance of lowering protein intake in virtually all patients with advanced CKD [17]. In this context, attention has shifted to how to accomplish this goal and what strategies should be implemented. Previous Italian experiences have highlighted the role of protein-free food and the importance of offering patients a choice of diets [5,8,11,18,23,36,37]. Protein-free food is an important support for protein restriction in Italy, where these products are totally or partially reimbursed. However, these products are not available in most other European countries and it is felt that cultural or compliance barriers may limit interest in using them [22,38]. In this regard, we felt that testing patients’ attitudes would provide insights into the potential candidates for dietary management and their overall willingness to change dietary habits for health-related reasons. 

The key point of our exploratory, real-life study was the assessment of the readiness to change of a population affected by advanced CKD, who was asked to participate in a trial of protein-free food, which had not previously been tested. The population is characterized by a high median age, both reflecting the local CKD population, whose median age at start of dialysis is above 70 years overall, and the setting of care, since the unit gathers patients with advanced CKD, particularly if at high morbidity, notably cardiovascular, or in the context of “conservative” pre-dialysis care. 

The interest in this study is both general and specific. In a general sense, our study highlights patients’ interest in changing their habits for a health goal and their ability to make the change. In a specific sense, our study helps to identify strategies for introducing protein-free food in the diets of a cohort of patients with different dietary habits. In this respect, the stepwise comparisons discussed here support the decisional pathway. 

Not all patients are good candidates for a diet trial. In our cohort, about one-fourth of our patients (22.28%) were not considered for participation, due to short life expectancy (10/49), imminent dialysis start (8/49), cognitive impairment (13/49) or other barriers (18/49). This information may be relevant in clinical practice, as the new KDOQI guidelines clearly underline that the patients considered for a protein restriction approach should be metabolically stable, a condition that does not encompass short life expectancy and imminent dialysis start [17]. Additionally, our study may suggest that cognitive impairment and logistic barriers may also be relevant in this regard. 

However, the vast majority of our patients (77.73%) were considered potential candidates and in fact 80.70% of them agreed to participate in the study. In the context of a largely elderly, high-comorbidity population, patients who did not wish to test the new products were 10 years older (83 (55–103) years of age vs. 73 (25–97), *p* < 0.001), and consequently had higher CCI and MIS scores (CCI: eight (3–13) vs. seven (2–17); MIS four (0–12) vs. five (1–12)) than those who decided to participate, showing that baseline characteristics, specifically age, often modulate attitudes towards dietary choices.

The test was generally well rated by those who participated. The vast majority of patients were ready to change their previous eating habits by integrating low-protein products into their diets, and only about a quarter (25.38%) of the patients were reluctant to change, indicating on the satisfaction questionnaire that they would be willing to add the products in the test to their diet 0–3 times per week. In keeping with the importance of individual food preferences, no difference was detected between patients according to the frequency that they were ready to use the products they had tested.

An element of reflection is that pasta, the hallmark of the Italian cuisine, was one of the best-liked foods, a sign of patients’ willingness not only to accept a “healthy product”, but also their openness to different traditions. Food is a part of group identity and membership; however, with globalization, people are learning to appreciate new foods [39,40]. In fact, Italian pasta was one of the foods French patients, despite their strong cultural identity, liked best (Figure 2).

This study, which is one of the first to explore attitudes to integrating new products in the daily diet of a large, elderly, high-comorbidity CKD cohort, has several limitations. It is a monocentric study and the center’s particular system of CKD care may have played a role in influencing patients’ choices. Moreover, it evaluates a test of short duration and has no follow-up; therefore, making it impossible to assess efficacy, compliance and “diet tiredness”.

The roles of the patient–physician and patient–dietitian relationships were not explored. This is probably an important point in motivating the patients towards testing a new approach, and eventually following it in the long term. Indeed, in our center, a previous study aimed at assessing compliance in a first cohort of patients highlighted that over 75% of the patients were able to attain the prescribed dietary goals, suggesting that a flexible system of care was well adapted to our population [31].

**Figure 3:**
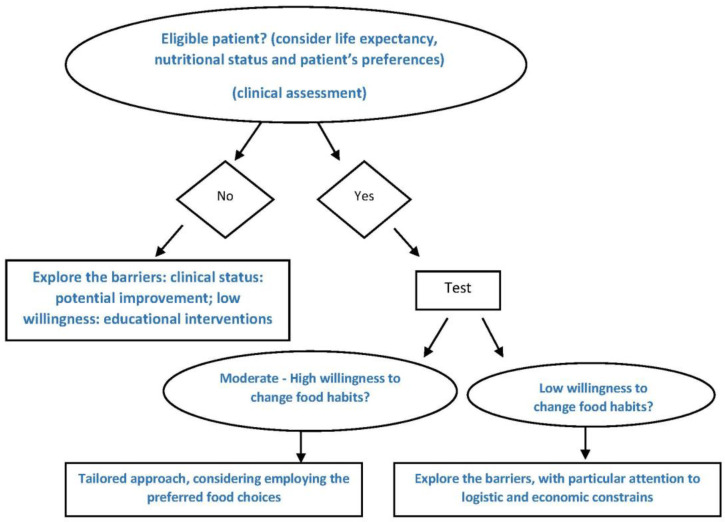
Tailored approach to dietary prescription.

Within these limits, our study shows that the “poor candidates” were a minority and may help identify a potential implementation strategy for “good candidates” (Figure 3). The overall readiness to change, which decreased as patients’ age increased, was found to be based on individual elements that cannot be determined in advance, as is witnessed by the wide and overlapping range of ages in both the cases of those who agreed to participate and those who did not. 

We suggest, therefore, that dietary options should be offered without preconceptions to all patients that could potentially benefit from them. In patients ready to test changes to their diets, the factor that made the difference regarding whether or not they would be willing to add the proposed protein-free food to their meals was taste. The lack of difference in baseline characteristics is in line with personal preference, and in keeping with the identification of taste as the main barrier to changing dietary habits. Therefore, our data strongly support that patients should be offered a test phase during which changes can be tailored to reflect their preferences. In our study, the products were given free of charge, all tests were reimbursed, as per the usual French policy, and indeed no biochemical test was added to the usual clinical management. However, logistic elements, most importantly reimbursement, were identified as important barriers to the integration of protein-free food in the long-term. This should be kept in mind when identifying implementation strategies, particularly when dealing with an elderly population, such as ours, in which economic hurdles are often relevant.

Our exploratory study was not designed for answering questions regarding adherence, or for assessing how much these products may help in reaching the targets recently reviewed and summarized in the KDOQUI guidelines [17]. This is a very important point for future studies; indeed, according to the Italian experience, there is no frequent “compensation” with protein rich food, since these products are rich in calories and, on the contrary, allow eating food of animal origin more freely. The main drawback in the Italian experience was low adherence, due to low palatability, which is one of the reasons why we designed this study [8,41].

Our study, the first one aiming to assess the potential for integrating protein-free food, so far essentially only used in Italy, in an elderly French population, may pave the way for multicentric studies aimed at defining the role and the room for these or other similar products to improve dietary adherence and reach the prescribed targets in different settings. Such a study could also highlight the roles of different cultural backgrounds and of the center organization in promoting adherence to the dietary prescriptions.

## 5. Conclusions

Our study suggests that CKD patients are keen to alter their diet to improve health related outcomes, and that even elderly and high-comorbidity patients are willing to change their dietary habits, although very old age (≥80 years) is associated with lower willingness to test new food. 

In patients who agreed to try the new products (protein-free food), the level of willingness to integrate them in their daily meals reflected individual preferences and taste, and thus underlined the importance of tailored approaches in improving adherence to protein-restricted diets in CKD patients. 

## Figures and Tables

**Table 1:** Baseline characteristics of the population.

	Proposed	Not Proposed	*p*	Accepted	Refused	*p*
*n*	171	49		138	33	
Gender, *n* (%)			**0.006**			0.861
males, *n* (%)	117 (68.42%)	23 (46.94%)		94 (68.12%)	23 (69.70%)	
females, *n* (%)	54 (31.58%)	26 (53.06%)		44 (31.88%)	10 (30.30%)	
Age, median (min–max)	77 (25–103)	79 (27–95)	0.156	73 (25–97)	83 (55–103)	**<0.001**
BMI (kg/m^2^), median (min–max)	28.25(17.43–51.20)	28.67(19.81–38.38)	0.978	28.73 (18.56–51.20)	26.56 (17.43–44.29)	0.051
Caucasian, *n* (%)	165 (96.49%)	47 (95.92%)	0.850	132 (95.65%)	33 (100%)	0.223
Charlson Comorbidity Index, median (min–max)	7 (2–17)	8 (2–14)	0.299	7 (2–17)	8 (3–13)	**0.008**
SGA score, *n* (%)			**<0.001**			**0.016**
SGA A	154 (90.06%)	31 (63.27%)		128 (92.75%)	26 (78.79%)	
SGA B	17 (9.94%)	17 (34.69%)		10 (7.25%)	7 (21.21%)	
SGA C	0	1 (2.04%)		0	0	
MIS score, median (min–max)	4 (0–12)	6 (1–13)	**<0.001**	4 (0–12)	5 (1–12)	**0.010**
sCreatinine (mg/dL), median (min–max)	2.14 (0.9–9.14)	2.30 (1.25–14.69)	0.204	2.18 (0.90–9.14)	1.91 (1.05–5.44)	0.055
eGFR-EPI (mL/min), median (min–max)	27 (6–68)	21 (3–59)	**0.021**	26.00 (6.00–60.00)	30.00 (7.00–68.00)	0.145
eGFR stage, *n* (%)			**<0.001**			**0.026**
Stage 3a *	14 (8.19%)	4 (8.16%)		7 (5.07%)	7 (21.21%)	
Stage 3b	59 (34.50%)	15 (30.61%)		49 (35.51%)	10 (30.30%)	
Stage 4	79 (46.20%)	13 (26.53%)		66 (47.83%)	13 (39.39%)	
Stage 5	19 (11.11%)	17 (34.69%)		16 (11.59%)	3 (9.09%)	
Proteinuria (g/day), median (min–max)	0.4 (<0.01–8.10)	0.3 (<0.01–6.02)	0.563	0.50 (<0.01–8.10)	0.20 (<0.01–3.10)	0.285
Diabetes, *n* (%)	73 (42.69%)	18 (36.73%)	0.455	60 (43.48%)	13 (39.39%)	0.670
Protein intake, *n* (%)			**<0.001**			0.200
Unrestricted, *n* (%)	0	12 (24.49%)		0	0	
0.8 g/kg/day, *n* (%)	102 (59.65%)	21 (42.86%)		78 (56.52%)	24 (72.73%)	
0.6 g/kg/day, *n* (%)	45 (26.32%)	9 (18.37%)		40 (28.99%)	5 (15.15%)	
0.6 g/kg/day supplemented, *n* (%)	24 (14.03%)	7 (14.29%)		20 (14.49%)	4 (12.12%)	

Note: statistically significant data in bold. Abbreviations: BMI: body mass index; SGA: subjective global assessment; MIS: malnutrition inflammation score; eGFR: estimated glomerular filtration rate; eGFR-EPI: estimated glomerular filtration rate according to the Chronic Kidney Disease Epidemiology Collaboration equation. * Stage 3a includes 2 patients in stage 2 that were previously in stage 3a and that subsequently had an improvement in their renal function.

**Table 2:** Baseline characteristics of the population according to low, moderate, and good willingness to change.

	Low Willingness to Change	Moderate Willingness to Change	High Willingness to Change	*p*
N 130	33 (25.38%)	49 (37.69%)	48 (36.92%)	
Gender, *n* (%)				0.987
males	22 (66.67%)	32 (65.31%)	32 (66.67%)	
females	11 (33.33%)	17 (34.69%)	16 (33.33%)	
Age, median (min–max)	77 (25–97)	72 (35–96)	74 (37–97)	0.897
BMI (kg/m^2^), median (min–max)	28.25 (20.31–51.20)	28.04 (18.56–51.17)	29.02 (19.33–44.20)	0.951
Caucasian, *n* (%)	32 (96.97%)	47 (95.92%)	45 (93.75%)	0.774
Charlson score, median (min–max)	7 (2–13)	7 (2–14)	7 (2–13)	0.746
SGA score, *n* (%)				0.999
SGA A	31 (93.94%)	46 (93.88%)	45 (93.75%)	
SGA B	2 (6.06%)	3 (6.12%)	3 (6.25%)	
MIS score, median (min–max)	4 (1–8)	4 (0–10)	3.5 (1–9)	0.129
sCreatinine (mg/dL), median (min–max)	2.13 (1.15–5.77)	2.40 (1.31–5.40)	2.13 (0.90–9.14)	0.824
eGFR-EPI (mL/min), median (min–max)	25 (7–58)	24 (10–55)	28 (6–60)	0.648
eGFR stages, *n* (%)				0.656
Stage 3a *	1 (3.03%)	4 (8.16%)	2 (4.17%)	
Stage 3b	14 (42.42%)	12 (24.49%)	18 (37.50%)	
Stage 4	15 (45.45%)	26 (53.06%)	23 (47.92%)	
Stage 5	3 (9.09%)	7 (14.29%)	5 (10.41%)	
Proteinuria (g/day), median (min–max)	0.20 (0–6.50)	0.80 (0.10–7.70)	0.35 (0–8.10)	0.111
Diabetes, *n* (%)	16 (48.48%)	22 (44.90%)	18 (37.50%)	0.586
Protein intake, *n* (%)				0.199
Unrestricted, *n* (%)	0	0	0	
0.8 g/kg/d, *n* (%)	24 (72.73%)	23 (46.94%)	27 (56.25%)	
0.6 g/kg/d, *n* (%)	7 (21.21%)	16 (32.65%)	14 (29.17%)	
0.6 g/kg/d, Ketosteril, *n* (%)	2 (6.06%)	10 (20.41%)	7 (14.58%)	
WHOQOL-BREF domains, median (min–max)				
Domain 1: physical health, median (min–max)	3.33 (2.17–4.00)	3.33 (2–4.33)	3.42 (2.14–4.71)	0.882
Domain 2: psychological, median (min–max)	3.67 (2.33–4.5)	4 (2–5)	4 (2.17–5)	0.557
Domain 3: social relationships, median (min–max)	3.67 (2.67–4.50)	4 (2–5)	4 (1.67–5)	0.619
Domain 4: environment, median (min–max)	3.63 (2.88–4.75)	3.63 (2.75–4.63)	3.82 (2.25–4.75)	0.790

Abbreviations: BMI: body mass index; SGA: subjective global assessment; MIS: malnutrition inflammation score; eGFR: estimated glomerular filtration rate; eGFR-EPI: estimated glomerular filtration rate according to the Chronic Kidney Disease Epidemiology Collaboration equation WHOQOL-BREF: World health organization quality of life questionnaire, short form. * Stage 3a includes 1 patient now in stage 2, previously in stage 3a. One questionnaire was too incomplete to be analyzed.

**Table 3:** Logistic regression analysis: outcome: refuse of a diet trial.

			95% Confidence Intervals		
		Odds-Ratio	Lower	Higher	*p*-Value
Step 1	Gender (females/males)	0.643	0.257	1.608	0.345
	Age (≥80 years)	5.408	1.894	15.447	**0.002**
	Charlson (≥7)	1.039	0.300	3.600	0.952
	SGA (B vs. A)	1.856	0.580	5.938	0.297
Step 2	Gender (females/males)	0.643	0.257	1.607	0.345
	Age (≥80 years)	5.503	2.263	13.384	**<0.001**
	SGA (B vs. A)	1.856	0.580	5.935	0.297
Step 3	Age (≥80 years)	5.308	2.197	12.826	**<0.001**
	SGA (B vs. A)	1.639	0.532	5.051	0.390
Step 4	Age (≥80 years)	5.891	2.527	13.737	**<0.001**

Note: statistically significant data in bold.

**Table 4:** Logistic regression analysis for low willingness to change.

			95% Confidence Intervals		
		Odds Ratio	Lower	Higher	*p*-Value
Step 1	Gender (females/males)	0.996	0.419	2.367	0.992
	Age (≥80 years)	0.814	0.296	2.239	0.690
	SGA (B vs. A)	1.043	0.177	6.135	0.963
	Charlson (≥7)	1.292	0.506	3.298	0.593
Step 2	Age (≥80 years)	0.814	0.298	2.223	0.687
	SGA (B vs. A)	1.041	0.183	5.935	0.964
	Charlson (≥7)	1.292	0.508	3.289	0.591
Step 3	Age (≥80 years)	0.819	0.313	2.140	0.684
	Charlson (≥7)	1.291	0.507	3.285	0.592
Step 4	Charlson (≥7)	1.180	0.513	2.714	0.696

Abbreviations: SGA: subjective global assessment.
